# Improvement in Essential Oil Quantity and Quality of Thyme (*Thymus vulgaris* L.) by Integrative Application of Chitosan Nanoparticles and Arbuscular Mycorrhizal Fungi under Water Stress Conditions

**DOI:** 10.3390/plants12071422

**Published:** 2023-03-23

**Authors:** Mostafa Amani Machiani, Abdollah Javanmard, Ali Ostadi, Khoshnood Alizadeh

**Affiliations:** 1 Department of Plant Production and Genetics, Faculty of Agriculture, University of Maragheh, Maragheh 55181-83111, Iran; 2 Dryland Agricultural Research Institute, Agricultural Research, Education and Extension Organization (AREEO), Maragheh 55176-43511, Iran

**Keywords:** bio-fertilizer, drought stress, medicinal and aromatic plants, secondary metabolites, thymol

## Abstract

Water stress is one of the critical abiotic stresses and limiting factors in the productivity of plants, especially in arid and semi-arid regions. In recent years, the application of bio-fertilizer and stress-modulating nanoparticles (NPs) is known as one of the eco-friendly strategies for improving plants quantity and quality under stressful conditions. In order to achieve the desirable essential oil (EO) quality and quantity of thyme in water deficit conditions, a 2-year field experiment was carried out as a split plot based on the randomized complete block design (RCBD), with 12 treatments and three replications. The treatments included different irrigation levels, containing irrigation at 80% field capacity (FC80) as no stress, 60% FC as moderate water stress (FC60) and 40% FC as severe water stress (FC40), as well as four different fertilizer sources, including non-application of fertilizer (control), application of arbuscular mycorrhizal fungi (AMF), chitosan NPs (CHT) and co-application of AMF+CHT NPs. The results demonstrated that the dry yield of thyme decreased by 13% and 40.3% under FC60 and FC40 water stress conditions. However, co-application of AMF+CHT NPs enhanced the dry yield of thyme by 21.7% in comparison to the control (non-application of fertilizer). The maximum EO content (2.03%) and EO yield (10.04 g 7 g m^−2^) of thyme were obtained under moderate water stress (FC60) fertilized with AMF+CHT NPs. Co-application of AMF+CHT NPs enhanced the EO content and EO yield of thyme by 17.1% and 42.7%, respectively. Based on the GC-MS and GC-FID analysis, 38 constituents were identified in the thyme EO, with the major constituents being thymol (35.64–41.31%), *p*-cymene (16.35–19.38%), *γ*-terpinene (12.61–13.98%) and carvacrol (2.78–3.93%) respectively. The highest content of thymol and *γ*-terpinene was obtained under moderate water stress (FC60) fertilized with AMF+CHT NPs. In addition, the highest content of *p*-cymene and carvacrol was observed in the severe water stress (FC40) fertilized with AMF+CHT NPs. The present research suggests that the co-application of AMF+CHT NPs represents a sustainable and eco-friendly strategy for improving the EO quantity and quality of thyme under water stress conditions.

## 1. Introduction

Medicinal and aromatic plants (MAPs) are able to synthesize a diverse group of secondary metabolites that are useful remedies for humans and also have great importance in several fields including pharmaceuticals, food flavor, perfumery, etc. [1]. Thyme (*Thymus vulgaris* L.) is an aromatic herb belonging to the family Lamiaceae with medicinal and functional properties, so it has attracted the attention of the food, cosmetic, perfumery and pharmaceutical industries [2,3]. Thyme, as traditional and folklore medicine, is used for the treatment of chest infections (bronchitis, pharyngitis, whooping cough), cough, diabetes, etc. [4]. Further, the volatile EO distilled from aerial parts of thyme has antibacterial, antispasmodic, antiseptic, antifungal and expectorant analgesic properties [5,6]. It has been reported that the thyme EO is rich in oxygenated (60% mixture of thymol and carvacrol, linalool) and hydrocarbon monoterpenoids (*p*-cymene and *γ*-terpinene) [7].

The increasing temperature as a result of greenhouse gas emissions led to an increase in drought stress and water shortage in the agricultural sector. In Iran, water stress plays a special role in reducing the yield of plants because the average annual rainfall in this country is 250 mm which is 70% less than the world average [8]. Water stress is one of the critical abiotic stresses and limiting factors in the productivity of plants, especially in arid and semi-arid regions. Water stress negatively affects plants morphological, physiological and biochemical properties. The adverse effects of water stress on plants include decreasing photosynthesis rate due to reduction of CO_2_ absorption, increased photorespiration, obstructed ATP synthesis and increasing membrane lipid peroxidation as a result of enhancement of the activity of reactive oxygen species (ROS), which, ultimately, decreases the quantity and quality of agricultural products [9].

In addition to the negative impacts of water stress on plants, nutrient accessibility is reduced in these conditions due to the reduction in mobility and low rate of mineral diffusion [10]. In this condition, the increasing consumption of chemical fertilizers to compensate for the efficiency of nutrients enhance the production costs of agricultural products. Therefore, the use of new and sustainable strategies for increasing the efficiency of nutrients in arid and semi-arid regions is very important. In recent years, the application of bio-fertilizer and stress-modulating nanoparticles (NPs) is known as one of the eco-friendly strategies for improving plant quantity and quality under stressful conditions [11].

Nanotechnology is a process of generating, manipulating and deploying nanomaterials into a system and is known as one of the novel and creative approaches in the agriculture section for modulating the negative impacts of stressful conditions and increasing nutrient uptake in these conditions [12,13]. This technology employs nanoparticles (NPs) having at least one dimension in the order of 100 nm or less [14]. NPs hold great promise regarding their application in agriculture in terms of plant protection and nutrition due to their size-dependent qualities, high surface-to-volume ratio and unique optical properties [15]. Among different NPs, CHT can play an important role in improving the quantity and quality of plant products under stressful conditions. CHT, as a major component of the shells of crustaceans such as crab, shrimp and crawfish, is a natural polysaccharide derived by the N-deacetylation of chitin [16]. CHT NPs and their derivatives are non-toxic, biodegradable and friendly to the environment and also have a great potential for agricultural application and enhancing crop production under stressful conditions through increasing the antioxidant enzyme activity that leads to decreases in the negative impacts of ROS compounds [17]. Additionally, previous reports showed that the application of CHT NPs in different plant species could improve plant performance by increasing macro- and micro-nutrient uptake, growth stimulator, germination acceleration, etc. [18].

In addition to nanoparticles, the use of beneficial soil organisms as bio-fertilizers is known as another sustainable solution for improving nutrient uptake and crop productivity under water stress conditions [19,20,21]. Bio-fertilizers contain beneficial bacteria and fungi that convert essential nutrients from inaccessible to accessible forms during biological processes [22]. Arbuscular mycorrhizal fungi (AMF), belonging to the *phylum Glomeromycota*, can form mutualistic symbiotic association with more than 80% of plant species. The symbiotic association of AMF with plant roots positively affects plant growth by increasing nutrient and water uptake, increasing photosynthetic rate by regulating the chloroplast enzyme activity, reducing chlorophyll decomposition rate, promoting chlorophyll synthesis and also increasing the tolerance of plants in different stressful conditions through the augmentation of antioxidant defense systems [23]. Additionally, AMF can reprogram the metabolic pathways of plants, resulting in changes in the primary and secondary metabolites. It has been reported that the inoculation of AMF improves the EO quantity and quality of different medicinal and aromatic plants, such as holy basil (*Ocimum tenuiflorum* L.), sage (*Salvia officinalis* L.), peppermint (*Mentha piperita* L.), thyme (Thymus vulgaris L.), tarragon (*Artemisia dracunculus*), lavender (*Lavandula angustifolia*), etc. [5,8,14,24,25].

Improving the quantitative and qualitative yield and essential oil of MAPs, especially in stressful conditions, increases the economic value of these plants. In conventional agricultural systems, increasing the yield of plants has a direct relationship with the excessive use of chemical inputs. It should be noted that the excessive use of chemical fertilizer, especially higher doses of N fertilization, decreases the yield of bioactive compounds in MAPs [26]. Therefore, the need to use new eco-friendly fertilizers to increase the efficiency of nutrients in water stress conditions and improve the quantitative and qualitative characteristics of medicinal and aromatic plants seems to be necessary. The literature is scant about the integrative application of bio-fertilizers along with stress-modulating NPs on the quantity and quality of MAPs essential oils. Therefore, the study aimed to investigate the effects of nano- and bio-fertilizer (separately and combined with each other) on the nutrient concentration, morphological and phytochemical characteristics of thyme under water stress conditions. We hypothesize that (i) the integrative application of nano- and bio-fertilizer enhance the nutrient concentration, (ii) increase thyme productivity and (iii) improve EO quantity and quality of thyme seedlings under water stress conditions.

## 2. Results

### 2.1. Analysis of Variance

The analysis of variance results showed that most of the studied traits (except canopy diameters) were significantly impacted by irrigation levels (I), fertilizer application (F) and the interaction of two mentioned factors (I × F). In addition, the canopy diameter of thyme was significantly impacted by different irrigation levels and fertilizer application and the interaction of two factors (I × F) was not significantly impacted in this trait (Table 1).

### 2.2. AMF Colonization

**Figure 1:**
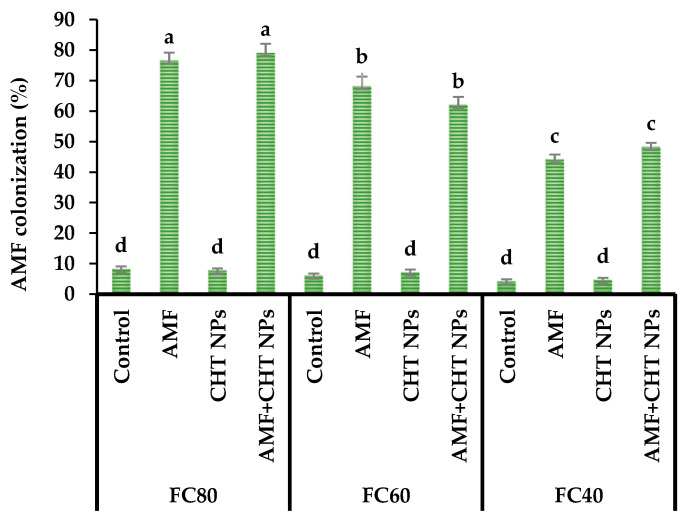
The AMF root colonization of thyme in different irrigation levels and fertilizer sources.

The highest AMF colonization percentage was obtained under well-watered conditions (FC80) treated with AMF+CHT NPs and a separate application of AMF. In comparison with well-watered conditions, the AMF colonization rate was reduced by 16.5% and 41.1% under moderate (FC60) and severe water stress (FC40), respectively (Figure 1).

### 2.3. Nutrient Concentration

The maximum concentration of N (22.31 g kg^−1^), P (2.49 g kg^−1^) and K (19.06 g kg^−1^) was achieved by integrative application of AMF+CHT under non-stress conditions (FC80). However, the minimum concentration of N (14.99 g kg^−1^), P (1.36 g kg^−1^) and K (11.49 g kg^−1^) was noted in the severe water stress conditions (FC40) without fertilization. The concentration of N, P and K was reduced by 10.7%, 22.8% and 15% in the FC60 water stress and reduced by 19.2%, 31.3% and 26.1% in the FC40 water stress, respectively. Interestingly, the co-application of AMF+CHT NPs enhanced the concentration of N, P and K of thyme by 16.9%, 19.5% and 18.6% in comparison with the non-application of fertilizer (Table 2).

### 2.4. Agronomic Traits

#### 2.4.1. Plant Height

The highest plant height of thyme (23.65 cm) was measured in non-stress conditions (FC80) treated with AMF+CHT NPs. Moreover, the lowest thyme height (15.96 cm) was found in the severe water stress (FC40) without fertilizer application. The height of thyme decreased by increasing water stress levels. The plant height in the FC60 and FC40 water stress decreased by 13.1% and 21.7% in comparison with non-stress conditions (FC80), respectively. Interestingly, the separate application of AMF and CHT NPs and co-application of AMF+CHT NPs enhanced thyme height by 7.2%, 3.7% and 14%, respectively (Table 3).

#### 2.4.2. Canopy Diameter

**Figure 2:**
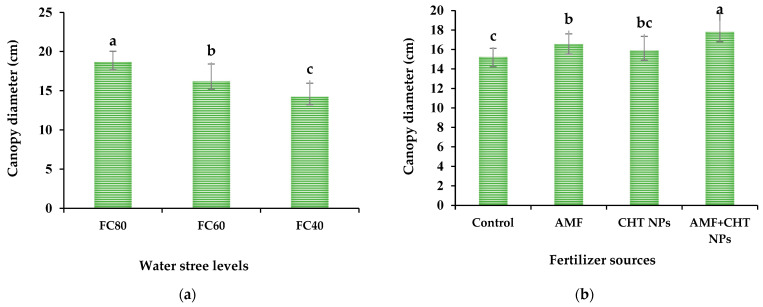
The canopy diameter of thyme under different water stress levels (**a**) and fertilizer sources (**b**).

Among different water stress levels, the highest (18.67 cm) and lowest (14.23 cm) canopy diameter of thyme was found in the non-stress (FC80) and severe water stress conditions (FC40). The canopy diameter of thyme decreased by enhancing water stress levels. The thyme canopy diameter in the FC60 and FC40 water stress conditions declined by 13.3% and 23.8% in comparison with non-stress conditions (FC80), respectively (Figure 2a). Additionally, the separate application of AMF and CHT NPs and co-application of AMF+CHT NPs enhanced thyme canopy diameter by 8.6%, 4.3% and 16.9%, respectively, when compared with the control (Figure 2b).

### 2.5. Fresh Yield

The integrative application of AMF+CHT NPs under no water stress conditions (FC80) produced the maximum fresh yield of thyme (1806.53 g m^−2^). In comparison with FC80, the fresh yield of thyme was reduced by 15.1% and 29.1% under FC60 and FC40 water stress levels. Additionally, the separate application of AMF and CHT NPs and co-application of AMF+CHT NPs increased thyme fresh yield by 11%, 5.9% and 19.8%, respectively, when compared with the control (Table 3).

### 2.6. Dry Yield

The integrative application of AMF+CHT NPs under no water stress conditions (FC80) produced the highest dry yield of thyme (570.4 g m^−2^). In comparison with FC80, the dry yield of thyme was reduced by 13% and 40.3% under FC60 and FC40 water stress levels. Additionally, the separate application of AMF and CHT NPs and co-application of AMF+CHT NPs increased thyme dry yield by 12.1%, 9.3% and 21.7%, respectively, when compared with the control (Table 3).

### 2.7. Essential Oil Content

The maximum EO content of thyme (2.03%) was obtained under moderate water stress (FC60) fertilized with AMF+CHT NPs. In contrast, the minimum EO content of this plant (1.34%) was obtained in the no water stress conditions (FC80) without fertilization. Under moderate (FC60) and severe water stress (FC40), the EO content of thyme increased by 33.6% and 24.5%, respectively. Interestingly, the separate application of AMF and CHT NPs and co-application of AMF+CHT NPs increased thyme EO content by 10.1%, 3.8% and 17.1%, respectively, when compared with the control (Table 3).

### 2.8. Essential Oil Yield

The maximum EO yield of thyme (10.04 g m^−2^) was obtained under moderate water stress (FC60) fertilized with AMF+CHT NPs. In contrast, the minimum EO yield of this plant (4.54 g m^−2^) was detected in the severe water stress conditions (FC40) without fertilization. In comparison with no water stress conditions (FC80), the EO yield of thyme was enhanced by 16.3%. Moreover, the separate application of AMF and CHT NPs and co-application of AMF+CHT NPs increased thyme EO yield by 22.7%, 13.1% and 42.7%, respectively, when compared with the control (Table 3).

### 2.9. Essential Oil Constituents

**Figure 3:**
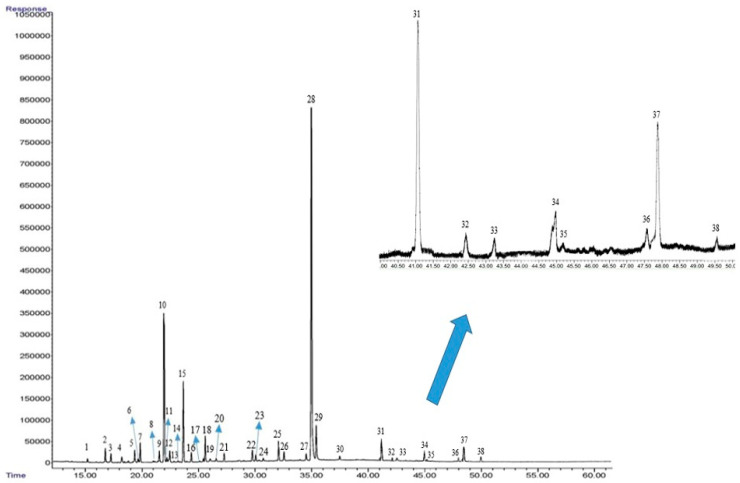
GC–FID chromatogram of thyme essential oils. The characterized peaks are numbered according to the constituent numbers in Table 4.

Based on the GC-MS and GC-FID analysis, 38 constituents were identified in the thyme EO, with the major constituents being thymol (35.64–41.31%), *p*-cymene (16.35–19.38%), *γ*-terpinene (12.61–13.98%) and carvacrol (2.78–3.93%) (Figure 3). The highest content of thymol and *γ*-terpinene was obtained under moderate water stress (FC60) fertilized with AMF+CHT NPs. Further, the highest content of *p*-cymene and carvacrol was observed in the severe water stress (FC40) fertilized with AMF+CHT NPs. The content of thymol, *p*-cymene and *γ*-Terpinene increased by 4.8, 6.4 and 3.3% under FC60 and increased by 3.2, 8.6 and 2.1% under the FC40 water stress level, respectively. Additionally, among different fertilizer sources, the co-application of AMF+CHT NPs had the greatest effect on the content of thymol, *p*-cymene and *γ*-terpinene and enhanced the content of the three mentioned constituents by 5.5%, 7.3% and 3.6%, respectively, when compared with the control (Table 4).

### 2.10. Correlation

**Figure 4:**
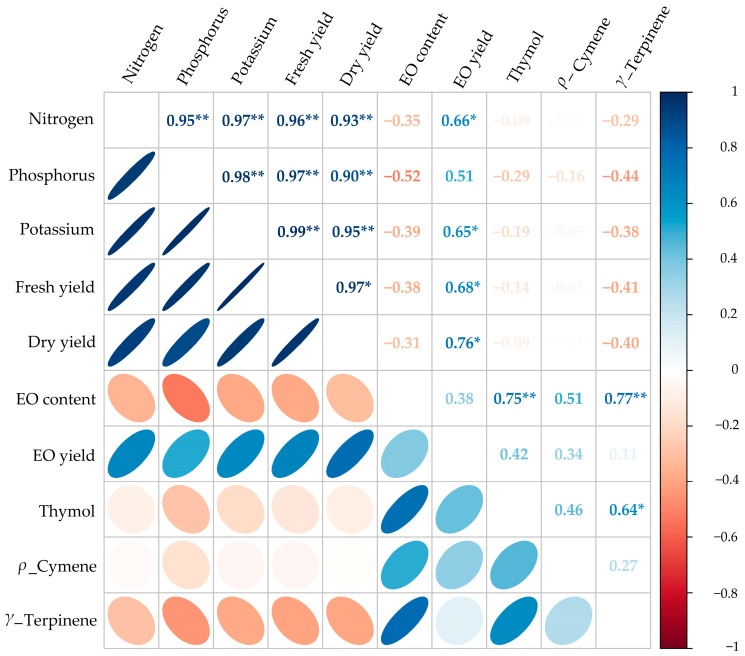
Pearson’s correlation matrix for studied traits of thyme. Color ellipses illustrate statistically significant levels. Positive and negative correlations are shown with blue and red, respectively. The color legend on the right-hand side of the corrplot represents the intensities of the Pearson correlation coefficients. * and ** indicate significant correlation at the 5% and 1% probability level, respectively.

The results showed that the dry yield of thyme had a significant positive correlation with fresh yield, EO yield and N, P and K content (r = 0.97, 0.76, 0.93, 0.90 and 0.95, respectively, *p*-value < 0.01). Likewise, EO content presented a positive and significant correlation with thymol and *γ*-Terpinene (r = 0.75 and 0.77, respectively, *p*-value < 0.01). Additionally, EO yield significantly correlated with N, P, fresh and dry yield (r = 0.66, 0.65, 0.68 and 0.76, respectively) (Figure 4).

## 3. Discussion

The results of this study demonstrated that the AMF colonization rate reduced under moderate and severe water stress conditions. The soil water availability played a key role in the soil microbial activity and had a negative impact on the AMF root colonization when the soil water was limited. Therefore, the reduction in root AMF colonization under water stress conditions could be attributed to a reduction in activity, spore germination and development of AMF [27]. In accordance with the results of the present study, Ostadi et al. [8] reported that the AMF colonization rate of peppermint decreased under moderate and severe water deficit conditions.

The nutrient uptake of thyme decreased under water stress conditions. The reduction of nutrient uptake under water stress conditions could be explained by decreasing nutrient supply through mineralization, decreasing mass flow and nutrient diffusion that affected the kinetics of nutrient uptake per unit of roots [28]. In addition, Sanaullah et al. [29] concluded that the nutrient solubility in the rhizosphere area affected by soil microbial activity, such as AMF, and under water limitation conditions decreased as a result of reducing the activity of the soil microbial population. It is worth noting that the nutrient concentration of thyme under well-watered and water stress conditions sharply increased by co-application of AMF+CHT NPs. It seems that the symbiotic association of AMF with thyme roots enhances nutrient concentration by increasing the absorption rate and area as a result of extensive underground extra-radical mycelia and also acidification of the rhizosphere area by the release of H^+^ [30]. Additionally, the higher nutrient content by CHT NPs application could be attributed to the higher surface area to the volume and slower diffusion of nanoparticles [31]. Dzung et al. [32] reported that the application of CHT enhanced the content of N, P, K and other micro-nutrients in the coffee plant. Furthermore, Sathiyabama and Manikandan [18] reported that foliar application of CHT NPs enhanced the mineral content of finger millet plants.

In this study, the agronomic traits and also the fresh and dry yield of thyme significantly reduced under moderate (FC60) and severe water stress (FC40). The reduction in the yield component as well as thyme productivity (fresh and dry yield) could be explained by the lower availability of sufficient moisture around the root zone and, thus, a lesser proliferation of root biomass, resulting in the lower absorption of nutrients and water, which have negative impacts on the photosynthesis rate, cell differentiation and division, and finally, decrease plant growth parameters and yield [10]. Similarly, Javanmard et al. [33] noted that the fresh and dry yield of Balamgu (*Lallemantia Iberica*) decreased by 14.9% and 15.3% under moderate water stress (FC60%) and decreased by 33.9% and 34.2% under severe water stress (FC30%), respectively. On the other hand, the co-application of AMF+CHT NPs improved thyme productivity under water stress conditions. The increasing thyme productivity could be explained by the role of the symbiotic association of AMF with thyme roots that enhance nutrient solubility as well as water uptake [10,24]. Additionally, the application of CHT causes tolerance to dehydration by reducing transpiration and maintaining the relative water content and also improving plant performance under stressful conditions through increasing antioxidant activity and decreasing the negative impacts of reactive oxygen species (ROS compounds) [34]. 

The results of this study showed that the EO content and main EO constituents of thyme, such as thymol, *p*-cymene and *γ*-terpinene increased in water stress condition. Unlike other plants where the economic value decreases under drought stress conditions, the economic value of MAPs under these conditions enhances due to the biosynthesis of secondary metabolites. The biosynthesis of secondary metabolites in MAPs is known as one of the defensive mechanism systems for increasing the adoption of these plants in the face of stressful conditions [35]. In drought stress conditions, the photosynthesis rate of plants decreased due to closing stomata and less absorption of CO_2_, which led to the accumulation of NADPH+H^+^ in plant cells. Therefore, the biosynthesis of secondary metabolites, such as EO compounds, alkaloids, phenolics, etc., improves plant efficiency through the consumption of NADPH+H^+^ [8]. 

Interestingly, the integrative application of AMF+CHT NPs improved EO productivity and EO quality of thyme by increasing the main essential oil constituents, such as thymol, *p*-cymene and *γ*-terpinene. It seems that the AMF-plant symbiotic association increased nutrient and water uptake, which led to an improved photosynthesis rate and improved the biosynthesis of secondary metabolites through the enhancement of intermediate (such as acetyl coenzyme A, NADPH and ATP) and EO precursor compounds, including pyruvate glyceraldehyde phosphate, erythrose-4-phosphate and phosphoenolpyruvate that are required for EO compounds biosynthesis [1]. In addition, the application of CHT NPs could decrease chlorophyll destruction by modulating the negative effects of drought stress. Increasing the chlorophyll activity under stressful conditions plays an important role in improving the photosynthesis rate and producing sufficient carbohydrates for cell growth and the production of EO-secreting glands, and in this way, it will be able to increase the EO content in the plant. In accordance with the results of the present study, Mazrou et al. [36] noted that the application of CHT NPs enhanced the EO content of *Matricaria chamomilla* L. by 57.14% and 47.06% during the two seasons of study. Further, Golubkina et al. [24] showed that the inoculation of AMF with *Artemisia dracunculus* and *Lavandula angustifolia* roots enhanced the EO content of both plants by 3% and 18% compared with the untreated control, respectively.

The highest EO yield of thyme was obtained under moderate water stress (FC60) fertilized with AMF+CHT NPs. Based on the correlation results, the EO yield of thyme calculated from dry yield multiplies with EO content and has a positive relationship with the two mentioned factors (Figure 4). Any factor that has a positive effect on the two mentioned factors ultimately enhances the EO yield in the MAPs. Therefore, increasing the EO yield of thyme could be explained by the enhancement of EO content under moderate water stress and integrative application of AMF+CHT NPs and also the positive role of the mentioned fertilizers on the thyme dry yield.

## 4. Materials and Methods

### 4.1. Study Site

The experiment was performed over two successive growing years (2020–2021) at the Maragheh University research farm, East Azerbaijan Province, Iran. The field soil of the experimental area had a sandy clay loam texture with a pH of 7.71. The soil’s physical and chemical properties are shown in Table 5. Further, the monthly weather data in the research area are provided in Table 6.

### 4.2. Experiment Design and Crop Management

The experiment was conducted with a split-plot based on the randomized complete block design (RCBD) with three replications. The treatments included different irrigation levels, consisting of irrigation at 80% field capacity (FC80) as no stress, 60% FC as moderate water stress (FC60) and 40% FC as severe water stress (FC40), as well as four different fertilizer sources, including non-application of fertilizer (control), application of arbuscular mycorrhizal fungi (AMF), chitosan NPs (CHT) and co-application of AMF+CHT NPs. Each plot contains 5 rows with a 40 cm distance between rows. Further, a 1.5 m distance was maintained between different treatments in order to exclude any influence of lateral water movement. Thyme Seedlings were obtained from Pakan Bazr company, Isfahan, Iran. The seedlings were sown with a density of 12.5 plants m^−2^ on 10 April 2020 and 15 April 2021, respectively. The first irrigation was performed immediately after sowing. The weeds were regularly controlled by hand. It is worth noting that the different water stresses were applied one month after sowing. In the AMF treatments, the *Funneliformis mosseae* fungus was isolated from the rhizosphere soil of the continuous cropping thyme. For mass production of *F. mosseae*, spores were propagated in sterilized soil, river sand and vermiculite cultured with alfalfa as a host plant for about 5 months in a greenhouse. AMF consists of *F. mosseae* colonized roots, spores, hyphae and substrate [37]. 

To reach chitosan nanoparticles, we tried to use a low molecular weight of chitosan (100 kD; Sabz Gostaresh Azin Turkan; Maragheh; Iran) with a degree of deacetylation of 85%. Tripolyphosphate (TPP; Merck company, Darmstadt, Germany), a gelling agent, was used as a crosslinker to attain chitosan nanoparticles. To reach homogeneous chitosan nanoparticles, we tried to use a procedure that was reported by Ahmadi et al. [38]. A CHT solution with a concentration of 0.1 wt% was obtained by dissolving 1 g of chitosan in 1000 mL of 0.1 wt% of acetic acid solution. After reaching a homogeneous solution, a TPP solution (0.4 g in 20 mL distilled water) was slowly dropped into the CHT solution while stirring vigorously. The formation of CHT NPs was evident from the appearance of a homogeneous cloudy solution. Finally, to adjust the pH of the chitosan nanoparticle solution at 7, 0.1 M of KOH was used. CHT NPs were sprayed two times in the seedling stage (one month after planting) with an interval of 10 days. For this purpose, CHT was dissolved in 3% (*v*/*v*) 0.1 M acetic acid by gentle heating at 60 °C and continuous stirring for 12 h, and then the pH was adjusted to 5.6 using 1 M NaOH solution. Finally, the CHT NPs were dissolved in sterile, distilled water and sprayed on thyme plants at a concentration of 0.5% *w*/*v*. After the preparation of the synthesized chitosan nanoparticles, the characterization of the nanoparticle was examined.

### 4.3. Measurements

#### 4.3.1. Fresh and Dry Yield

At full flowering stage, the thyme aerial parts were harvested randomly on 20 August 2020 and 25 August 2021, respectively. The plants were harvested randomly from a 1.6 m^−2^ area (middle line of each treatment). After weighing the fresh yield of each treatment, the samples were dried at room temperature for two weeks, and the dry matter yield was recorded.

#### 4.3.2. Essential Oil Content, Yield and Constituents

The EO content of thyme was extracted by hydro distillation method. Briefly, 40 g of dry matter of aerial parts of thyme was mixed with 400 mL water and heated for 3 h by a Clevenger-type apparatus. The extracted EO was dehydrated with anhydrous sodium sulfate and kept at 4 °C. The EO content and EO yield of thyme were calculated with the following equations [39]:

Essential oil content = (Extracted essential oil/40 g dry matter) × 100(1)

Essential oil yield (g m^−2^) = Essential oil content × dry yield (2)

The EO constituents of thyme were evaluated by Agilent 7990 B, USA gas chromatograph linked to a 5988A mass spectrometer with HP-5MS capillary column that features including: 5% phenylmethyl polysiloxane, length 30 m, 0.25 mm inner diameter and 0.25 μm film thickness. Briefly, the initial temperature of the column was programmed at 60 °C (5 min), then directly increased to 240 °C at the rate of 30 °C/min and held for 20 min. Helium was used as the carrier gas at a flow rate of 1 mL/min. The injector was set up as split ratio (1:30). The injector, detector and ionization temperatures were programmed to 230, 240 and 220 °C, respectively. The electron impact (EI) and mass scan range were 70 eV, and 40–400 *m*/*z*, respectively. To calculate the linear retention indices (RIs), a mixture of homolog series of n-alkanes was injected into the GC instrument based on the above analytical conditions. The identification process was performed using an interactive combination of chromatographic linear retention indices, determined by reference to a homologous series of n-alkanes (C8-C30) (Sigma, St. Louis, MO, USA) that were consistent with those reported in the literature [40]. For non-polar stationary phases, we used MS data consisting of the computer matching with the WILEY275, NIST 05, ADAMS and homemade (based on the analyses of reference oils and commercially available standards) libraries [40,41,42,43]. The EO constituents were separated using an Agilent 7990B gas chromatography machine (Santa Clara, CA, USA) linked with a capillary column VF-5MS with 30 m length, 0.25 mm internal diameter, 0.50 μm film thickness, 5% phenyl methylpolysiloxane and a flame ionization detector (FID). The column temperature was planned based on the same as the above analytical conditions. The essential oil was diluted in n-hexane solution (1:100) and injected with volume of 1 µL. Further, the percentage compositions of essential oil calculated were based on peak area normalization. For the calculation of the retention index (RI) of each constituent, the following equation was used [44]:

RI (i) = (100*n*) + [(100 × (ln (RT (i)) − ln (RT_inf_ (i))/(ln (RT_suo_ (i)) − RT_inf_ (i))] (3)

In this equation, n is the number of carbon atoms in n-alkane that eluted immediately before the i-th peak. RT (i) is the retention time (in minutes) of the i-th peak, RT_inf_ (i) is the reference n-alkane retention time that eluted before the i-th peak, and ln (RT_suo_ (i) is the retention time of (n + 1)- alkane that eluted after the i-th peak. 

#### 4.3.3. Nutrient Content

For measuring the nutrient (P and K) concentration of the thyme plant, 1 g of dry matter of aerial parts was poured into a porcelain crucible and heated in a muffle furnace. The temperature of the muffle furnace slowly increased to 550 °C and stayed there for 6 h until white ash was formed. After the muffle furnace cooled, the ash samples dissolved in 5 mL HCL 2N at room temperature for 15 min. Then, the samples were filtered through filter paper and brought to a volume of 50 mL and kept for 30 min. The K and P content was estimated by a flame photometer (Jenway PFP7/C, Keison Products, Chelmsford, United Kingdom) and yellow color method using a spectrophotometer at 470 nm (UV-1800, Shimadzu, Kyoto, Japan), respectively [45]. In addition, the concentration of nitrogen was determined by the Kjeldahl method [46].

#### 4.3.4. Root AMF Colonization

To determine the root colonization rate, first, root samples were cut into pieces of 1 cm and then rinsed with water until the extra particles were eliminated. To remove the cytoplasmic contents from cells, root samples were heated in 10% KOH for 15 min. After three times washing with distilled water, root pieces were acidified in 2% HCL for 10 min. After that, the root samples were placed in the hot 0.05% trypan blue solution for 10 min and finally washed with distilled water [47,48]. Finally, the root colonization rate was determined using the gridline intersect method that was proposed by Giovannetti and Mosse [49].

### 4.4. Statistical Analysis

Before the analysis of variance, the normality and homoscedasticity of the data were carried out using the Kolmogorov–Smirnov and Levene test, respectively. All obtained data were analyzed by SAS (SAS Institute Inc., Cary, NC, USA) software and the mean comparisons were analyzed by the least significant difference (LSD) test at the 95% level of probability. In addition, R software (v4.1.3) was used to draw the correlation plot (Pearson correlation matrix); the studied traits include nutrient concentration (N, P and K), fresh and dry yield, EO content, EO yield and main EO constituents (thymol, *p*-cymene and *γ*-terpinene).

## 5. Conclusions

The results of the study exhibit that the agronomic traits and dry yield of thyme seedlings reduced under moderate and severe water stress conditions. However, in comparison with well-watered conditions, the essential oil productivity enhanced under moderate and severe water stress conditions. Further, in both well-watered and water stress conditions, the co-application of AMF+CHT NPs increased dry yield, essential oil content and improve essential oil quality of thyme by increasing the main essential oil constituents, such as thymol, *p*-cymene and *γ*-terpinene. Generally, it can be concluded that the integrative application of biofertilizer (AMF)+ chitosan nanoparticles could be recommended as a sustainable and eco-friendly strategy for improving essential oil quantity and quality of thyme under water stress conditions.

## Figures and Tables

**Table 1:** The ANOVA (*p*-value) results of nutrient content, agronomic traits, fresh yield, dry yield, essential oil content and yield of thyme affected by experimental factors.

Source of Variation	Root Colonization	N Content	P Content	K Content	Plant Height	Canopy Diameter	Fresh Yield	Dry Yield	EO Content	EO Yield
I	<0.0001 **	<0.0001 **	<0.0001 **	<0.0001 **	<0.0001 **	<0.0001 **	<0.002 **	<0.005 **	<0.0001 **	<0.009 **
F	0.018 *	<0.0001 **	<0.0001 **	<0.0001 **	<0.0001 **	<0.0001 **	<0.003 **	<0.002 **	<0.0001 **	<0.002 **
I × F	<0.0001 **	0.006 **	<0.0001 **	0.002 **	<0.0001 **	0.845 ^ns^	0.004 **	0.002**	<0.0001 **	0.001 **

I: irrigation levels; F: fertilizer sources. ns, * and ** indicate no significant difference, significant at the 5% probability level and significant at the 1% probability level, respectively.

**Table 2:** The concentration of nitrogen (N), phosphorus (P) and potassium (K) in thyme at different irrigation levels and fertilizer sources.

Treatments		N (g kg^−1^)	P (g kg^−1^)	K (g kg^−1^)
FC80	Control	19.39 cd	2.09 b	16.36 bc
	AMF	20.11 bc	2.19 b	17.03 b
	CHT	20.98 b	2.18 b	16.99 b
	AMF+CHT	22.31 a	2.49 a	19.06 a
FC60	Control	17.25 fg	1.61 def	13.86 ef
	AMF	18.23 def	1.80 c	15.16 cde
	CHT	18.65 de	1.68 cde	14.35 de
	AMF+CHT	19.84 bc	1.83 c	15.68 bcd
FC40	Control	14.99 h	1.36 g	11.49 g
	AMF	16.62 g	1.56 ef	12.82 fg
	CHT	17.07 fg	1.49 fg	12.36 g
	AMF+CHT	18.2 ef	1.74 cd	14.70 de
LSD_0.05_		1.16	0.166	1.36

Different letters indicate significant differences at the 5% level according to LSD’s test.

**Table 3:** The plant height, fresh yield, dry yield, essential oil content and yield of thyme in different irrigation regimes and fertilizer sources.

Treatments		Plant Height (cm)	Fresh Yield (g m^−2^)	Dry Yield (g m^−2^)	Essential Oil Content (%)	Essential Oil Yield (g m^−2^)
FC80	Control	20.95 bcd	1535.69 cd	480.12 cd	1.34 g	6.43 ef
	AMF	22.27 ab	1703.50 ab	536.05 ab	1.39 g	7.45 de
	CHT	21.58 bc	1632.50 ab	545.72 ab	1.39 g	7.58 de
	AMF+CHT	23.65 a	1806.53 a	570.4 a	1.58 f	9.01 abc
FC60	Control	18.33 fg	1328.23 fg	434.02 d	1.75 de	7.58 de
	AMF	19.63 def	1457.56 ef	477.13 cd	1.99 ab	9.49 ab
	CHT	18.82 ef	1376.96 ef	450.27 cd	1.85 bcd	8.33 bcd
	AMF+CHT	20.15 cde	1510.44 cde	494.76 bc	2.03 a	10.04 a
FC40	Control	15.96 h	1049.43 h	273.55 f	1.66 ef	4.54 h
	AMF	17.29 gh	1181.61 gh	317.60 f	1.83 cd	5.81 fg
	CHT	16.83 h	1135.52 h	302.24 f	1.67 ef	5.05 gh
	AMF+CHT	19.17 ef	1369.85 ef	380.35 e	1.95 abc	7.42 de
LSD_0.05_		1.48	154.04	52.64	0.14	1.28

Different letters indicate significant differences at the 5% level according to LSD’s test.

**Table 4:** The essential oil constituents of thyme in different irrigation regimes and fertilizer sources.

No	Components	LRI Exp. ^a^	LRI Lit. ^b^	FC80 Control	FC80 AMF	FC80 CHT	FC80 AMF+CHT	FC60 Control	FC60 AMF	FC60 CHT	FC60 AMF+CHT	FC40 Control	FC40 AMF	FC40 CHT	FC40 AMF+CHT	ID ^c^
1	Tricyclene	920	921	0.09	0.02	0.11	0.06	0	0.03	0.07	0.05	0	0	0.03	0.07	RI-MS
2	*α*-Thujene	929	930	1.66	1.34	1.43	1.52	1.33	1.49	1.94	1.78	1.7	1.65	1.76	2.03	RI-MS
3	*α*-Pinene	931	932	1.21	0.56	0.49	0.54	0.68	0.55	1.06	0.84	0.86	1.21	0.59	0.92	Std
4	Camphene	944	946	0.88	0.65	0.41	0.84	0.78	0.59	0.92	1.04	0.42	0.65	0.37	0.44	Std
5	Sabinene	968	969	1.1	0.73	0.68	0.85	0.71	0.89	1.04	0.98	0.78	0.82	0.83	0.68	Std
6	1-Octen-3-ol	972	974	0.79	0.63	0.66	0.5	0.46	0.6	0.7	0.61	0.61	0.48	0.38	0.42	RI-MS
7	*β*-Myrcene	985	988	2.09	1.83	1.96	2.02	1.88	2.12	1.98	2.21	1.79	2.08	1.98	2.16	Std
8	3-Octanol	997	998	0.05	0.06	0	0.09	0	0.04	0.03	0	0.05	0.04	0.03	0	RI-MS
9	*α*-Terpinene	1011	1014	1.22	1.18	1.02	1.21	1.08	1.15	1.27	1.3	1.28	1.25	1.16	1.33	RI-MS
10	*p*-Cymene	1019	1020	16.35	16.72	18.3	18.05	17.68	18.84	18.73	18.5	18.19	19.32	18.34	19.38	Std
11	Limonene	1022	1024	0.56	0.39	0.18	0.41	0.23	0.29	0.46	0.52	0.19	0.27	0.23	0.37	RI-MS
12	1,8-Cineole	1024	1026	3.02	3.15	2.89	2.63	3.18	3.26	2.89	2.63	3.69	3.18	1.85	2.63	Std
13	Z-*β*-Ocimene	1030	1032	0.08	0.09	0.02	0	0.02	0	0.03	0	0	0.03	0.04	0.02	Std
14	E-*β*-Ocimene	1042	1044	1.12	0.78	0.39	0.49	0.77	0.61	0.42	0.39	0.86	0.42	0.44	0.69	Std
15	*γ*-Terpinene	1052	1054	13.11	12.83	13.12	12.61	12.87	13.7	12.83	13.98	13.07	12.46	13.29	13.89	Std
16	*cis*-Sabinene hydrate	1064	1065	0.63	0.21	0.35	0.36	0.44	0.51	0.41	0.27	0.54	0.47	0.51	0.29	RI-MS
17	*α*-Terpinolene	1066	1068	0.04	0.06	0.08	0.08	0.09	0.11	0.06	0.05	0.07	0.06	0.06	0.08	RI-MS
18	Linalool	1094	1095	2.06	2.09	1.83	2.16	2.18	1.54	1.97	2.07	1.84	2.09	2.15	1.37	Std
19	*α*-Campholenal	1124	1126	0	0	0	0.01	0	0	0	0	0	0.01	0	0	RI-MS
20	*trans*-Sabinol	1135	1137	0.02	0.01	0	0	0.02	0.03	0.01	0	0	0.01	0.03	0	RI-MS
21	Camphor	1137	1141	1.54	1.06	1.28	1.46	1.03	1.51	0.75	1.09	1.04	0.6	1.31	1.75	RI-MS
22	Borneol	1161	1165	0.8	0.7	0.45	0.96	0.72	0.82	0.57	0.72	0.54	0.81	0.7	0.53	Std
23	Terpinen-4-ol	1171	1174	0.62	0.54	0.47	0.18	0.52	0.4	0.32	0.46	0.7	0.38	0.28	0.45	RI-MS
24	*α*-terpineol	1193	1195	0.07	0.05	0.02	0.03	0	0.05	0.05	0	0.08	0.02	0.02	0.04	RI-MS
25	Neral	1234	1235	0.06	0.08	0.04	0.04	0.03	0.07	0.02	0.03	0.03	0.05	0.06	0.04	Std
26	Carvacrol, methyl ether	1238	1241	1.39	1.17	1.06	1.25	1.1	0.73	1.46	1.37	0.89	1.33	1.13	1.03	RI-MS
27	Geranial	1260	1264	0.33	0.31	0.31	0.28	0.27	0.29	0.32	0.33	0.34	0.31	0.28	0.29	RI-MS
28	Thymol	1287	1289	35.64	38.74	38.15	37.87	37.99	39.55	38.91	41.31	38.25	39.3	38.86	39.02	Std
29	Carvacrol	1297	1298	2.94	2.99	3.19	3.32	3.23	3.35	3.7	2.78	3.35	3.07	3.03	3.93	Std
30	Thymol acetate	1347	1349	0.71	0.53	0.61	0.88	0.68	0.4	0.31	0.34	0.35	0.56	0.58	0.59	RI-MS
31	(*E*)-Caryophyllene	1415	1417	3.23	3.55	3.48	2.29	3.22	2.4	2.29	2.05	2.9	2.27	2.85	3.38	Std
32	*α*-Humulene	1451	1452	0.06	0	0	0.04	0	0	0	0.05	0	0.04	0	0	RI-MS
33	Linalool isovalerate	1463	1466	0.07	0.11	0.06	0.03	0.04	0.03	0	0.02	0	0.03	0.03	0.05	RI-MS
34	Germacrene D	1481	1484	0.48	0.25	0.53	0.41	0.31	0.34	0.38	0.4	0.23	0.58	0.51	0.39	RI-MS
35	Bicyclogermacrene	1498	1500	0.03	0	0	0.02	0	0.02	0	0	0.04	0.03	0	0	RI-MS
36	*δ*-Cadinene	1521	1522	0.23	0.36	0.39	0.23	0.15	0.18	0.09	0.15	0.39	0.28	0.42	0.39	RI-MS
37	Caryophyllene oxide	1581	1582	0.69	0.58	0.72	1.22	1.16	1.02	0.67	0.55	0.32	0.91	0.83	0.64	Std
38	*β*-Bisabolene	1766	1768	0	0	0	0.03	0	0.05	0	0	0.04	0	0	0.03	RI-MS
	Total identified (%)			94.78	94.16	95.49	94.78	94.66	97.37	96.47	98.68	95.24	96.88	94.77	99.06	

^a^ LRI, linear retention indices, experimentally determined using homolog series of *n*-alkanes. ^b^ Relative retention indices taken from Adams. ^c^ Identification methods: MS, by comparison of the mass spectrum with those of the computer mass libraries Wiley, Adams and NIST 08; RI, by comparison of retention index with those reported in the literature; Std, by comparison of the retention time and mass spectrum of available authentic standard.

**Table 5:** Physico-chemical properties of field soil (average of two years).

Soil Texture	Sand (%)	Silt (%)	Clay (%)	OM (g kg^−1^)	EC (ds.m^−1^)	pH	FC (%)	PWP (%)	N (g kg^−1^)	P (mg kg^−1^)	K (mg kg^−1^)
Sandy clay loam	56.1	17	26.9	8.2	1.16	7.71	26.8	13.7	0.85	9.8	549.34

OM: organic matter, FC: field capacity, PWP: permanent wilting point.

**Table 6:** Monthly average temperature and total monthly precipitation in 2020 and 2021 growing seasons and long-term averages in the experimental area.

Year	April	May	June	July	August	September
Monthly average temperature (°C)						
2020	11.8	19.1	24.2	28.0	25.1	23.8
2021	16.3	21.3	27.2	28.3	28.1	23.02
2-year mean	14.1	20.2	25.7	28.1	26.6	23.4
10-year mean	12.9	18.5	24.4	28.1	27.5	22.7
Total monthly precipitation (mm)						
2020	63.3	12.0	2.6	0.1	1.2	0.0
2021	12.01	13.3	0.01	3.10	0.02	0.1
2-year mean	37.6	12.7	1.3	1.6	0.6	0.05
10-year mean	41.8	19.9	1.5	0.7	0.3	1.8

## Data Availability

The datasets generated and analyzed during the current study are available from the corresponding author upon reasonable request.
